# The Theta Rhythm of the Hippocampus: From Neuronal and Circuit Mechanisms to Behavior

**DOI:** 10.3389/fncel.2021.649262

**Published:** 2021-03-04

**Authors:** Angel Nuñez, Washington Buño

**Affiliations:** ^1^Departamento de Anatomía, Histología y Neurociencia, Facultad de Medicina, Universidad Autonoma de Madrid, Madrid, Spain; ^2^Instituto Cajal, Consejo Superior de Investigaciones Cientificas, Madrid, Spain

**Keywords:** hippocampal neurons, medial septum, diagonal band of Broca, cholinergic input, NMDA, gamma oscillations

## Abstract

This review focuses on the neuronal and circuit mechanisms involved in the generation of the theta (θ) rhythm and of its participation in behavior. Data have accumulated indicating that θ arises from interactions between medial septum-diagonal band of Broca (MS-DbB) and intra-hippocampal circuits. The intrinsic properties of MS-DbB and hippocampal neurons have also been shown to play a key role in θ generation. A growing number of studies suggest that θ may represent a timing mechanism to temporally organize movement sequences, memory encoding, or planned trajectories for spatial navigation. To accomplish those functions, θ and gamma (γ) oscillations interact during the awake state and REM sleep, which are considered to be critical for learning and memory processes. Further, we discuss that the loss of this interaction is at the base of various neurophatological conditions.

## Introduction

The hippocampus is the main structure involved in the generation of the 4- to 12-Hz theta (θ) rhythm, which is one of the most regular EEG oscillation that can be recorded from the mammalian brain. Interest in θ has flourished from the abundant data indicating that the rhythm is linked with integrative processes critical for higher cognitive functions. Thus, neuronal spiking in widespread brain regions is phase locked to hippocampal θ oscillations, including somatosensory, entorhinal, or prefrontal cortices (Alonso and Garcia-Austt, 1987; Nuñez et al., 1991, 2006; Kocsis and Vertes, 1994; Hanada et al., 1999; Sirota et al., 2008).

A slow oscillatory activity with the properties of θ was first described in the hippocampus by Jung and Kornmüller (1938). However, the original detailed analysis of hippocampal θ was provided by Green and Arduini (1954). Green and Arduini (1954) showed that θ was associated with an irregular desynchronized activity in the cortex, whereas in contrast, synchronized cortical activity was concurrent with irregular activity in the hippocampus. This link between hippocampal and cortical activities suggested a close relation of θ with attention, information processing, higher brain functions, and cognition, giving rise to a rapidly growing interest in θ. Indeed, more than 2,000 articles that mention hippocampal θ have been published from 1950 to 2020, with more than 50 articles published in 2020.

Although more than 150 reviews on hippocampal θ have been published, several aspects of the neuronal and circuit mechanisms involved in the generation of the rhythm and particularly its participation in behavior remain unknown or controversial. Nevertheless, new experimental data and modern interpretation of former results provide insight to those unresolved issues. Therefore, this review focuses on those debated basic mechanisms of θ genesis and of its relationships with behavior. However, it is of key importance to consider that “after 50 years and hundreds of experiments, there is no widely accepted term that would unequivocally describe behavioral correlate(s) of hippocampal θ oscillation” (Buzsaki, 2020). Moreover, the physiological inputs triggering the selective activation of hippocampal pyramidal neurons in natural conditions are undefined. Therefore, a large amount of future imaginative research will be required to unravel the functional correlation between behavior and hippocampal θ.

## Medial Septum–Diagonal Band of Broca and Theta

A key contribution was the discovery by Petsche et al. (1962) and Stumpf et al. (1962) of the role of the medial septum (MS) in controlling the activity of the hippocampus and in generating θ. It has been shown that glutamatergic, cholinergic, and GABAergic MS and diagonal band of Broca (DbB) neurons project to the hippocampus (Monmaur and Thomson, 1983; Colom et al., 2005; Desikan et al., 2018; Unal et al., 2018). Indeed, evidence has accumulated suggesting that θ arises from interactions between MS-DbB and intra-hippocampal neuronal and circuital oscillators (Stumpf et al., 1962; McLennan and Miller, 1974; Stewart and Fox, 1989; Hangya et al., 2009; Huh et al., 2010; Müller and Remy, 2018). Strong support to the above interpretation was provided by lessions of the MS-DbB that blocked hippocampal θ and rhythmic neuronal activity (Green and Arduini, 1954; Buño et al., 1978; Mitchell et al., 1982). However, the rules of innervation of hippocampus by diverse MS-DbB neurons are unknown, and the contribution of hippocampal neuronal populations and circuits in the genesis of θ are a matter of debate.

**Figure 1:**
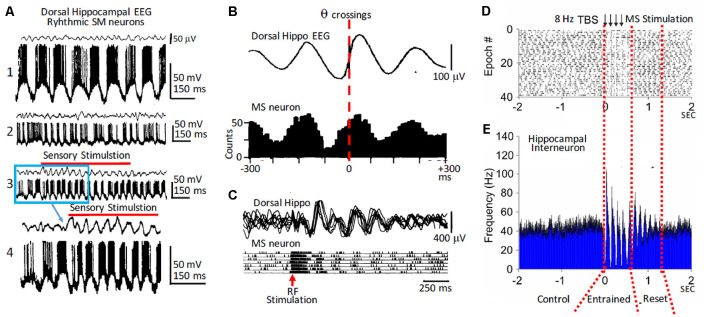
Rhythmic medial septum-diagonal band of Broca (MS-DbB) neurons and theta (θ): *in vivo* recordings. **(A1)** Intracellular recordings of a bursting neuron with large spontaneous θ-like membrane potential oscillations (lower) in the absence of θ in the hippocampal EEG (upper). **(A2)** As **(A1)**, but irregular bursting and θ. **(A3)** Change to higher amplitude θ and higher frequency rhythmic bursting induced by sensory stimulation (gentle stroking the animal’s back; red horizontal bar). **(A4)** Expanded version of part of **(A3)**; (blue square). **(B upper)** Cross-correlation function (triggered by θ zero crossings, interrupted red line) of hippocampal EEG. **(B lower)** Cross-correlation histogram of rhythmic busting medial septum (MS) neuron activity showing strong phase locking between both activities. Note the phase advance of the neuron with θ. **(C)** Reset of hippocampal θ (upper) and of rhythmic bursting MS neuron (lower) triggered by reticular formation stimulation (red arrow); notice the increased amplitude of reset θ. **(D)** Raster plot showing successive epochs of rhythmic bursting hippocampal interneuron before, during, and after theta burst electrical stimulation of the MS. **(E)** Cross-correlation histogram showing entrainment by stimulation followed by phase reset (interrupted red lines). Modified from: **(A)** Barrenechea et al. (1995); **(B,C)** Gaztelu and Buño (1982); **(D,E)** Mamad et al. (2015).

Paired recordings of MS and dorsal hippocampal neurons together with recordings of the hippocampal field activity in anesthetized rats suggested that rhythmic hippocampal neuronal activity and field θ depended upon the rhythmic activity of MS neurons (Macadar et al., 1970; Roig et al., 1970). Importantly, field θ and rhythmic activity of hippocampal neurons was only present when MS neurons fired rhythmically. However, a group of MS neurons fired rhythmic bursts in the absence of hippocampal θ. Therefore, rhythmicity in the MS can be independent from the hippocampal θ, but the hippocampal θ rhythm narrowly depends on MS neuron rhythmicity (Figures 1A,B). The rhythmic MS neurons that fire both with or without field θ could play a leading role in triggering the rhythmic activity of other MS neurons leading to the generation of hippocampal θ. In addition to the MS-DbB neurons that fire rhythmic bursts, there were also non-rhythmic MS-DbB neurons (Buño et al., 1978; Gaztelu and Buño, 1982; Alonso and Garcia-Austt, 1987; Barrenechea et al., 1995; Pedemonte et al., 1998).

The analysis of septo-hippocampal pairs revealed three main behaviors: (i) when both neurons were rhythmic (θ-pairs), they fired bursts phase locked with the θ waves and showed rhythmic cross-correlation histograms; (ii) pairs with a rhythmic and a non-rhythmic neuron (mixed pairs) showed periodic cross-correlation histograms, and both neurons showed firing relationships with the field θ; and (iii) finally, non-rhythmic pairs were uncorrelated and did not show a relationship with field θ. Importantly, similar types of rhythmic and of non-rhythmic neurons with and without a relationship with θ were found in the hippocampus (see the “Phase Reset of Theta” section below).

Simultaneous intracellular recordings of MS-DbB neurons and of the hippocampal field activity in anesthetized rats revealed neurons that showed large continuous periodic membrane potential (Vm) oscillations and action potential bursts (i.e., intracellular θ; Pedemonte et al., 1998). These neurons displayed essentially identical activity both in the absence or in the presence of field θ, suggesting that they played a key role in hippocampal θ genesis. Other neurons that showed intracellular θ could sporadically fire high amplitude slow spikes and action potential bursts. A third group displayed lower amplitude intracellular θ and briefer bursts. These two last groups only showed rhythmicity during field θ.

Using a similar experimental design, Barrenechea et al. (1995) showed that neurons in the lateral septum could also display intracellular θ and fire in close relation to the field θ. Taken together, the above results suggest that hippocampal θ oscillations critically depend on the rhythmic neuronal activity of the MS-DbB. The functional role of the non-rhythmic septo-hippocampal neurons that fire in relation with the θ rhythm and the network mechanism that underlie this unexpected correlation are intriguing and remain to be determined. Accordingly, these neurons, although non-rhythmic, carry information about θ oscillations. Tentatively, the non-rhythmic neurons could evoke transient changes of the rhythm such as phase reset (see the “Phase Reset of Theta” section below).

### The Cholinergic Medial Septum–Diagonal Band of Broca Input

Initially, it was proposed that the combined effects of a background excitatory influence provided by cholinergic MS-DbB inputs interacting with a precisely timed rhythmic inhibition provided the main excitatory and inhibitory inputs required to generate θ (Andersen and Eccles, 1962; Petsche et al., 1968; Smythe et al., 1992; Desikan et al., 2018). Data in support of the cholinergic contribution to hippocampal θ are: (i) increased release of acetylcholine (ACh) during θ (Dudar, 1977); (ii) induction of θ by ACh and by cholinergic agonist (Alonso and Garcia-Austt, 1987; MacVicar and Tse, 1989; Bland and Colom, 1993); (iii) production of θ by the increased cholinergic activity caused by blockade of ACh hydrolysis (Macadar et al., 1970; Roig et al., 1970; Buño et al., 1978; Gaztelu and Buño, 1982); and (iv) blockade of θ by specific cholinergic antagonists (Monmaur and Thomson, 1983; Vinogradova, 1995; Li et al., 2007; McQuiston, 2010).

Interestingly, ACh is released by cholinergic MS-DbB terminals in the interstitial space where it is hydrolyzed by acetylcholinesterase, suggesting a gradual protracted activation by “ambient” ACh (McCormick and Prince, 1986; Descarries et al., 2004; Ovsepian et al., 2004; Yamasaki et al., 2010; Domínguez et al., 2014). The induction of θ by ACh is mediated predominantly by muscarinic receptors (mAChRs) expressed in hippocampal pyramidal neurons (Monmaur and Thomson, 1983; Ovsepian et al., 2004; Fernández de Sevilla et al., 2020). In addition, the activity of GABAergic interneurons is controlled both through mAChR and nicotinic receptors (nAChRs) by a cholinergic input from the basal forebrain, suggesting that nAChRs also contribute in the genesis of θ (Frotscher and Léránth, 1985; Gray et al., 1996; Griguoli et al., 2010; Drever et al., 2011; Griguoli and Cherubini, 2012; Yakel, 2012; Cheng and Yakel, 2015).

The cholinergic effects on septo-hippocampal activity are behavior dependent, since selective optogenetic activation of cholinergic MS-DbB neurons triggered strong network effects during inactive behavioral states and weak effects during active behaviors in rats (Mamad et al., 2015). In addition, non-selective MS-DbB theta-burst electrical stimulation increased field θ synchronization and power, reset hippocampal rhythmic bursting neurons, entrained hippocampal place cells, and tuned the spatial properties (Figures 1D,E). Furthermore, optogenetic activation of cholinergic MS-DbB neurons at θ frequencies increased the power but caused poor entrainment of the hippocampal θ, an effect that was behavior dependent since it was stronger under anesthesia compared to awake mice (Vandecasteele et al., 2014). Taken together the above findings support the idea that when cholinergic neurons are highly activated during active exploration, additional activation has a reduced effect on hippocampal oscillations.

### The GABAergic Medial Septum–Diagonal Band of Broca Input

The contribution of rhythmic GABAergic MS-DbB neurons in the generation of θ has been firmly established (Andersen and Eccles, 1962; Smythe et al., 1992; Joshi et al., 2017; Unal et al., 2018). Rhythmic MS-DbB interneurons express parvalbumin and the hyperpolarization-activated cyclic nucleotide-gated (HCN) channels, which can contribute to their oscillatory properties (Varga et al., 2008; Hangya et al., 2009). Importantly, rhythmic GABAergic MS-DbB neurons mainly contact hippocampal inhibitory interneurons of all types that exert a strong rhythmic inhibition upon pyramidal neurons (Freund, 1992; Smythe et al., 1992; Hangya et al., 2009; Unal et al., 2018), thereby contributing to θ *via* rhythmic disinhibition (Tóth et al., 1997). Although debated, the firing synchronization provided by electrical synapses through gap junctions between inhibitory interneurons could also contribute to the generation of the θ rhythm (Konopacki et al., 2014; Posłuszny, 2014; Schoenfeld et al., 2014).

Recent evidence obtained with optogenetic entrainment at θ frequencies of specific GABAergic MS neurons in behaving mice revealed a vital involvement of these neurons in the generation and entrainment of the hippocampal field θ and of rhythmic action potential bursting of hippocampal neurons, whereas optogenetic silencing of these neurons strongly reduced field θ (Bender et al., 2015; Boyce et al., 2016; Gangadharan et al., 2016). The entrainment was not modified behavior, suggesting that although the synchronization of GABAergic MS neurons plays a vital role in the generation of θ, the rhythmically entrained circuits do not participate in behavior. Although the hippocampal field could follow the optogenetic activation of GABAergic MS neurons at θ frequencies, hippocampal neurons could also discharge at higher frequencies (Zutshi et al., 2018). The different behaviors of the field and neuronal activities suggest that GABAergic MS neurons trigger circuit mechanisms that accelerate the rhythmicity of hippocampal neurons. Indeed, MS GABAergic neurons regulate circuit activity *via* rhythmic disinhibition of pyramidal neurons (Freund, 1992; Smythe et al., 1992; Tóth et al., 1997; Buzsaki, 2002; Unal et al., 2012, 2018). These studies provide strong support to the notion that septal GABAergic projections regulate the hippocampal field potential oscillations *via* θ hippocampal interneurons.

### The Glutamatergic Medial Septum–Diagonal Band of Broca Input

Although excitatory MS-DbB inputs had previously been shown to participate in the production of θ (Núñez et al., 1987; Núñez et al., 1990b; Leung and Shen, 2004; Colom et al., 2005; Huh et al., 2010; Khakpai et al., 2016), recent experimental evidence has provided strong support to the contribution of glutamatergic MS-DbB neurons in the genesis of hippocampal θ. Accordingly, optogenetic activation of specific glutamatergic excitatory MS-DbB neurons both *in vitro* and in behaving animals showed that those neurons provided a critical contribution to the genesis of hippocampal θ, mainly through local modulation of septal neurons (Robinson et al., 2016). Huh et al. (2010), using both *in vitro* slice and septo-hippocampal preparations, reported that activation of identified glutamatergic MS-DbB neurons led to fast AMPA-mediated synaptic responses in hippocampal pyramidal neurons. In addition, activation of MS-DbB neurons with NMDA microinjections induced rhythmic bursting at θ frequencies both in hippocampal and MS-DbB neurons (see “Intrinsic Pyramidal Neuron Properties and Theta” and “Intrahippocampal Circuits and Theta” sections below).

## Intrinsic Pyramidal Neuron Properties and Theta

### *In vivo* Studies

**Figure 2:**
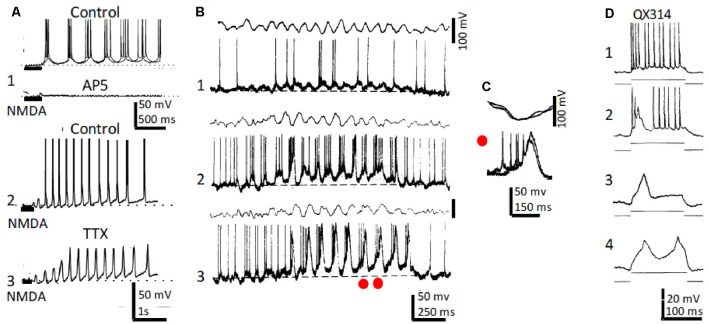
Rhythmic CA1 pyramidal neurons and theta (θ): *in vitro* and *in vivo* recordings. **(A1)** Representative *in vitro* patch recordings showing θ–like membrane potential oscillations and action potential bursts induced by NMDA microiontophoresis at the apical dendrites of a CA1 pyramidal neuron (upper); superfision of AP5 blocked the NMDA response (lower). **(A2)** As **(A1)**, but another neuron showing the effects of NMDA microiontophoresis in control ringer. **(A3)** Superfusion of TTX abolished action potential bursting but oscillations, NMDA- and Ca^2+^-spikes remained. **(B1)** Representative *in vivo* recordings of spontaneous CA1 EEG (upper) and CA1 neuron showing typical θ oscillations and single and occasional action potential bursts (lower). **(B2,3)** Same as **(B1)** in a CA3 neuron showing slow spikes riding on a sustained depolarization and firing rhythmic action potential bursts time-locked with θ oscillations; the red dots in **(A3)** correspond to the superimposed expanded records in panel **(C)**. **(D1)** Representative *in vivo* tonic discharge evoked by a depolarizing current pulse applied immediately after impalement with a QX314-filled electrode. **(D2)** Record obtained later after impalement showing a slow putative Ca^2+^ spike and fewer and smaller action potentials. **(D3,4)** Even later, action potentials were blocked and one and two slow spikes were triggered with increasing pulse intensities (1–4 same neuron). **(A)** Modified from Bonansco and Buño (2003); **(B,C)** modified from Núñez et al. (1987); **(D)** modified from Nuñez and Buño (1992).

Although the activity of the septo-hippocampal pathway and intrahippocampal circuits provide an important contribution to θ genesis, data has accumulated suggesting that intrinsic properties of hippocampal neurons also participate. Indeed, slow spikes were recorded in CA3–CA1 neurons of anesthetized rats together with the usual Na^+^ type action potential bursts during field θ. Slow spikes fired an overriding high-frequency burst of fast Na^+^-mediated action potentials (Núñez et al., 1987; Núñez et al., 1990a; Figures 2B,C). Depolarizing pulses triggered rhythmic slow spikes at rates that increased with depolarization. In the absence of field θ, strong dendritic depolarization by current injection induced large amplitude oscillation in the θ frequency range and resulted in a voltage-dependent phase precession of the action potentials as occurs in behaving animals (Kamondi et al., 1998).

In similar *in vivo* conditions, inhibition of Na^+^ conductance by QX-314-loading prevented fast Na^+^-mediated action potentials, but slow QX-314-resistant putative Ca^2+^ spikes remained (Nuñez and Buño, 1992; Figure 2D). In terms of their possible participation in the generation of θ, the slow QX-314-resistant events display the correct frequency and duration and can oscillate intrinsically. Accordingly, rhythmic bursts at θ frequency, similar to those triggered with intracellular recorded slow spikes, have been recorded *in vivo* in hippocampal neurons during periods of θ rhythm induced by sensory stimulation in anesthetized rats (Núñez et al., 1987) or when head-restrained mice, running on a spherical treadmill, entered in the recorded cell’s place field (Harvey et al., 2009). Accordingly, slow spikes provide pyramidal neurons with intrinsic conductance, which can boost θ oscillations generated by network properties.

### *In vitro* Studies

Recordings of CA1 pyramidal neurons in rat hippocampal slices revealed that plateau potentials and rhythmic θ-like intrinsic Vm oscillations were evoked by depolarizing current pulses in an important proportion of the neurons (García-Muñoz et al., 1993). Rhythmic high threshold slow TTX-resistant spikes were triggered and entrained by imposed sinusoidal transmembrane currents at θ frequencies. The above findings suggest that NMDA- and Ca^2+^-mediated spikes provide an important depolarizing drive, that boosts the otherwise small depolarization supplied by EPSPs, to generate the high-frequency rhythmic bursts of fast Na^+^-mediated action potentials that typifies pyramidal neuron activity during field θ.

In addition to the slow NMDA- and Ca^2+^-mediated spikes, voltage-gated Na^+^-mediated conductance underlie subthreshold rhythmic membrane oscillation in entorhinal cortex neurons, which could play an important part in the genesis of the θ rhythm (Alonso and Llinas, 1989; Dickson et al., 2000). These subthreshold Na^+^-dependent oscillations can also be recorded from CA1 hippocampal pyramidal neurons, suggesting a more direct contribution to hippocampal θ oscillations (García-Muñoz et al., 1993).

## Intrahippocampal Circuits and Theta

### Analysis *In vivo*

Due to the small amplitude of unitary EPSPs that on the average do not reach the firing threshold of hippocampal pyramidal neurons (Sayer et al., 1989; Fernández de Sevilla et al., 2002), action potential firing in CA1 pyramidal neurons is scarce during desynchronized hippocampal field activity (Schwartzkroin, 1975; Buzsaki and Eidelberg, 1983; Núñez et al., 1987; Csicsvari et al., 1999). However, during θ, there is strong increase in synchronized rhythmic activity of MS-DbB neurons that results in temporal and spatial summation of excitatory postsynaptic potentials (EPSPs) that exceed the firing threshold of hippocampal pyramidal neurons. The excitatory input of MS-DbB neurons onto hippocampal pyramidal neurons is paced by the rhythmic GABAergic inhibitory postsynaptic potentials (IPSPs) from both inhibitory MS-DbB and intrahippocampal interneurons. Interestingly, different interneuron types innervate distinctive domains of pyramidal neurons and exhibit specific firing patterns during θ, contributing differentially to hippocampal θ and ripple oscillations (Klausberger et al., 2003). The diversity of hippocampal interneurons could coordinate the activity of pyramidal cells in different behavioral states. Accordingly, the rhythmic interactions between EPSPs and IPSPs generate the intracellular θ and action potential bursting that typifies pyramidal neurons during θ (Andersen and Eccles, 1962; Núñez et al., 1987; Núñez et al., 1990a; MacVicar and Tse, 1989; Fujita, 1991; Cobb et al., 1995; Csicsvari et al., 1999).

Infusion of NMDA in the entorhinal cortex (Leung and Shen, 2004; Gu et al., 2017) and microiontophoresis of NMDA close to the apical dendrites of hippocampal pyramidal neurons in anesthetized rats induced the generation of hippocampal θ, demonstrating the involvement of glutamatergic NMDA receptors in intrahippocampal circuit activity (Puma et al., 1996; Bland et al., 2007). In addition, the hippocampal θ rhythm was markedly reduced by infusion of the specific NMDAR blocker AP5 into the lateral ventricles of behaving rats (Leung and Desborough, 1988). Therefore, the glutamatergic MS-DbB neurons can contribute to field θ acting through NMDARs in hippocampal neurons.

### The Theta Rhythm *In vitro*

Hippocampal oscillations frequencies within and exceeding the θ range can be induced *in vitro* by changes of the ionic environment, activation of ionotropic, and of metabotropic receptors. Superfusion of ACh muscarinic agonists *in vitro* can induce the intracellular θ and action potential bursting that typifies CA1 pyramidal neuron activity during θ oscillations in the natural condition (Konopacki et al., 1987; Bland et al., 1988; Fernández de Sevilla et al., 2006). The muscarinic rhythm is paced by local inhibitory interneurons, which are connected through dendritic electrical synapses and tend to fire synchronously (Traub et al., 2004; Konopacki et al., 2014; Posłuszny, 2014; Schoenfeld et al., 2014). Electrical coupling between pyramidal neuron axons have also been reported to contribute (Traub et al., 2004). Superfusion of NMDA in hippocampal slices induces θ-like oscillations, suggesting an important contribution of circuital excitatory synaptic interaction through NMDARs in the genesis of θ (Kazmierska and Konopacki, 2013).

Tetanic stimulation of Schaffer collaterals (SCs) and microiontophoresis of glutamate at CA1 pyramidal neuron apical dendrites evoked rhythmic Vm oscillations and action potential bursts at θ frequencies *in vitro* (Bonansco et al., 2002). Oscillations were clear cut in pyramidal neurons placed close to the midline of the dorsal CA1, but not in lateral neurons that fired single-action potentials. Medial neurons exhibited a higher NMDAR density at the apical dendritic shafts than lateral neurons and a larger NMDA current component under voltage-clamp, suggesting that these differences underlie the dissimilar responses of both neuron groups.

NMDA microiontophoresis at the apical dendrites of CA1 pyramidal neurons induced θ-like Vm oscillations and rhythmic action potential bursts *in vitro* (Bonansco and Buño, 2003). However, in the absence of NMDAR activation, imposed membrane depolarization and microiontophoresis of AMPA depolarized, but never induced, rhythmic oscillations and bursts. Rhythmic Vm oscillations and bursts induced by NMDA remained under blockade of GABA-mediated inhibition with picrotoxin and of AMPA receptors with CNQX. In contrast, oscillations and bursts were prevented by inhibition of NMDARs with AP5 and in Mg^2+^-free solutions. Importantly, NMDAR-mediated Vm oscillations persisted, but action potentials were prevented under blockade of Na^+^-mediated action potentials with tetrodotoxin (Figure 2A).

Taken together, the above results suggest that Vm oscillations in hippocampal CA1 pyramidal neurons induced by microiontophoresis of NMDA, glutamate, and by tetanic stimulation of SCs do not depend on circuital interactions. The results suggest that NMDA-induced oscillations relied on the negative slope conductance of the NMDA channel caused by the voltage-dependent Mg^2+^ block that underlies NMDA spikes (Schiller and Schiller, 2001; Antic et al., 2010) and on high-threshold Ca^2+^ spikes mediated by activation of L-type voltage-dependent Ca^2+^ channels (VDCC; Bonansco and Buño, 2003). The large Ca^2+^-mediated depolarization triggers the high-frequency action potential burst that backpropagates into the apical dendrites of pyramidal neurons inducing a supralinear Ca^2+^ influx into spines that can induce long-term synaptic plasticity (Lisman, 2017; Sakmann, 2017; Fernández de Sevilla et al., 2020).

## Phase Reset and Entrainment of Theta

To be considered an oscillator, a neuron or network must be intrinsically rhythmic, and the rhythm must be acceptably regular. Neural oscillators display two distinctive behaviors when perturbed by brief inputs, namely, phase reset and entrainment (e.g., Winfree, 1977; Barrio and Buño, 1990; McClellan and Jang, 1993). Phase reset and entrainment result from the continuously varying excitability of the neural oscillator during the oscillation cycle. In spiking and bursting neural oscillators, the phase is the normalized time since the last action potential. In field recordings of neural oscillations, the phase is the normalized time between successive peaks or successive troughs of the oscillation.

### Phase Reset of Theta

Phase reset is evoked by stimulating an input (i.e., a perturbation) and computing the phase shift or reset of the perturbed rhythm. The perturbation phase-locks the oscillation and results in periodic averages and cross-correlations of the rhythmic field and action potential activity (Figure 1C). Phase reset of θ and rhythmical hippocampal units can be induced by electrical stimulation of hippocampal afferents both in anesthetized and in behaving rats, and during rapid eye movement (REM) in sleep (García-Sánchez et al., 1978; Gaztelu and Buño, 1982; Lerma and García-Austt, 1985; Alonso et al., 1987; Núñez et al., 1990b; Vinogradova, 1995; Givens, 1996; McCartney et al., 2004; Jackson et al., 2008). Importantly, reset was consistently evoked by stimulation of structures with rich contacts with the MS-DbB, but abolished by destruction of the MS and fornix, suggesting that it could be induced by reset of MS-DbB neurons triggered by input from connected structures (Buño et al., 1978; Brazhnik et al., 1985). An alternative possibility is that direct inhibition of MS neurons by synchronized hippocampal output originally elicited by septal inputs may be the reset mechanism. Indeed, the genesis and tuning of the 0 rhythm is a complex process in which feedback control of the septal pacemaker by hippocampal rhythmic neurons is an important process (Alonso et al., 1987; Müller and Remy, 2018).

Stimulation of areas connected to the MS-DbB could elicit reset with higher frequencies than the spontaneous θ (Buño et al., 1978; García-Sánchez et al., 1978; Gaztelu and Buño, 1982; Alonso et al., 1987; Núñez et al., 1990b), suggesting independent θ generator systems, which can produce different rhythms (Kramis et al., 1975). Electrical stimulation of structures projecting to MS-DbB tended to induce phase reset of the field θ and of the rhythmic bursting neuronal activity. Remarkably, in close agreement with the expected behavior of coupled oscillators, afferent stimulation also resets the MS-DbB rhythmic neuronal activity, resulting in phase reset of both septal and hippocampal oscillations (Gaztelu and Buño, 1982; Alonso et al., 1987; Barrenechea et al., 1995; Pedemonte et al., 1998; Figure 1C). Accordingly, phase reset of θ has been found during conditioning, operant behavior, and special navigation (see the “Memory and Theta” section below).

### Entrainment of Theta

Entrainment also results from the phase sensitivity of neural oscillators and follows the rules of phase reset typified by phase locking (Winfree, 1977; Barrio and Buño, 1990; McClellan and Jang, 1993; Lakatos et al., 2019). Periodic electrical stimulation of the MS-DbB and lateral septum between 4 and 12 Hz evoked 1:1 entrainment and reset of field θ and phase locking of action potential bursts in hippocampal interneurons in behaving rats (Mamad et al., 2015; Figures 1D,E). Stimulation beyond the θ range evoked bursts at higher or lower frequency than stimulation frequencies, suggesting that the septo-hippocampal circuitry is tuned to oscillate in the θ range (Brazhnik et al., 1985; García-Muñoz et al., 1993).

Interestingly, in agreement with the important influence of inhibition in θ genesis, reset and entrainment can be induced by activation of individual GABAergic interneurons in hippocampal slices, a mechanism that can synchronize the firing of pyramidal cells (Cobb et al., 1995). Phase reset and entrainment links external stimuli with neuronal rhythmic events (Lakatos et al., 2019). This link has been analyzed in a simple neural pacemaker where it enables the detection of specific input characteristics that depend on the properties and frequency of the oscillator (Buño et al., 1984).

## Hippocampal Theta Rhythm and Behavior

Among the many functions that have been attributed to hippocampal θ, we will center on the relationships with locomotion, memory, and spatial navigation (Cherubini and Miles, 2015). A growing number of studies suggest that θ may represent a timing mechanism where hippocampal pyramidal neurons fire with a higher probability at the phase of the θ cycle when excitation by MS-DbB glutamatergic neurons added with the sustained excitation supplied by cholinergic inputs is maximal, and periodic perisomatic inhibition is minimal (Núñez et al., 1990b; Freund and Buzsaki, 1996; Csicsvari et al., 1999; Klausberger and Somogyi, 2008; Müller and Remy, 2018; see the “Analysis *In vivo*” section above). This timing system may represent a general mechanism devised to organize into temporal series, fragmented by θ oscillations in a moment-by-moment basis of movement sequences, memory encoding, and planned trajectories for spatial navigation.

### Locomotion and Theta

The correlation between locomotion and the amplitude and frequency of the hippocampal θ have suggested that movement sequences could be regulated by the θ oscillations (Whishaw and Vanderwolf, 1973; Buño and Velluti, 1977; Vanderwolf et al., 1977; Fuhrmann et al., 2015; Lu et al., 2020). Running speed in behaving animals shows a close correlation with the amplitude and frequency of the θ oscillations (Rivas et al., 1996; Ahmed and Mehta, 2012; Bender et al., 2015; Lu et al., 2020). Likewise, movements tend to occur at a specified phase of the θ oscillation (Buño and Velluti, 1977; Semba and Komisaruk, 1978; Joshi and Somogyi, 2020).

**Figure 3:**
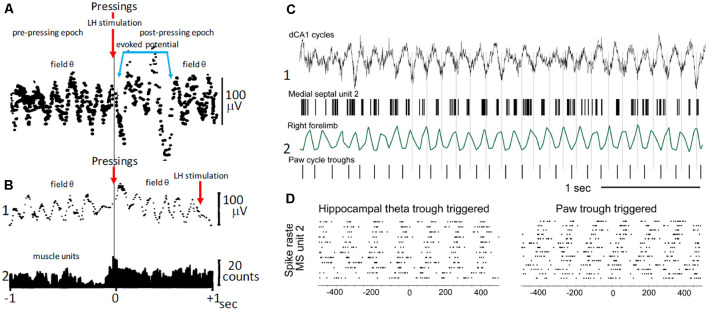
Voluntary movement and θ. **(A)** Phase relationship of θ with bar pressings for lateral hypothalamic (LH) electrical self-stimulation. The two superimposed averages triggered by bar pressing onsets (upper arrow) show θ waves in the pre-pressing epoch and a potential evoked by the LH self-stimulation (blue lines) followed by θ waves in the post-pressing epoch. **(B1)** As in **(A)**, average triggered by bar pressing onsets (lower left arrow) but the LH self-stimulation was delayed (0.9 s) from pressings onsets (lower right arrow). Note the absence of evoked potential and pre- and post-pressing θ waves in **(B1)**. **(B2)** Histogram of muscle unit firing from the left forelimb triggered by pressing onsets. **(A,B)** Two different rats; averages were constructed with 50 successive lever pressings. **(C)** Phase relationship between MS neuron discharges, step-cycles, and field θ in mice. **(C1)** Field θ and MS neuron action potentials (upper and lower, respectively). **(C2)** Forelimb movements and paw steps (upper and lower, respectively). **(D)** Raster plot of MS neuron firings synchronized with θ cycles and with paw steps (left and right, respectively). **(A)** Modified from Buño and Velluti (1977); **(B)** modified from Joshi and Somogyi (2020).

In freely behaving rats, bar pressing for electrical self-stimulation of the lateral hypothalamus, averages of hippocampal field activity triggered by the onset of bar pressings, revealed periodic waves with frequencies within the θ band (5–8 Hz; Figures 3A,B; Buño and Velluti, 1977). The periodic waves in the pre-pressing epoch are only observed if bar pressings tend to occur during a particular phase of the θ wave, implying that θ is phase-locked before pressing onsets. Accordingly, the periodic averages suggest that bar pressing onsets occurred at a defined phase of the ongoing field θ waves. There were also phase-locked θ waves following pressings superimposed on a potential evoked by the hypothalamic electrical self-stimulation (Figure 3A). Introduction of a delay (0.9 s) between bar pressings and the electrical self-stimulation delayed the evoked potential, and averages showed phase-locked θ oscillations before and after bar pressings (Figure 3B1).

Electrolytic lesions of the septum or superior fornix abolished θ and significantly increased the frequency of bar pressing. Accordingly, the septo-hippocampal mechanisms, which generate θ are not necessary to maintain self-stimulation (Ward, 1960). However, the increased lever pressing rates following lesions suggest a possible participation in motor timing mechanisms through an inhibitory influence of the septum (Gage et al., 1978).

The above results taken together suggest that phase-locked θ may be a corollary of motor mechanisms and perhaps of the timing of motor sequences controlled by θ cycles. Phase-locked θ oscillations could contain predictive information about the planning of motor activity (i.e., the future) and of its execution on a moment-by-moment basis that ends when the goal is reached (Whishaw and Vanderwolf, 1973; Wyble et al., 2000; Fuhrmann et al., 2015; Wikenheiser and Redish, 2015). Interestingly, step-cycles during walking in mice show a temporal correlation with θ oscillations and with the firing of MS neurons, suggesting that rhythmic firing MS cells could coordinate θ and stepping-related locomotor activity (Joshi and Somogyi, 2020; Figures 3C,D).

These findings fit in with the argument that phase locking of θ oscillations could regulate the planning and execution of motor activity on a moment-by-moment basis, where information is fragmented and organized into temporal sequences by θ oscillations. Hippocampal θ phase locking may thus represent a general neurophysiological mechanism supporting memory formation. It has been proposed that hippocampal neurons operate with future, present, and past events in the θ cycle sequences that hold spatio-temporal representations (Cei et al., 2014). The phase relationship of bar pressings with θ and of the subsequent hypothalamic stimulation may result in synaptic plasticity that is favored during a specific phase of the θ cycle (Buño and Velluti, 1977; Huerta and Lisman, 1995; McCartney et al., 2004).

### Memory and Theta

Although it has been firmly established that hippocampal θ carries spatial information (see the “Spatial Navigation and Theta” section below), a growing number of studies indicate that non-spatial information can be also conveyed by the hippocampal θ rhythm, suggesting that θ operates as a general mechanism for encoding continuous, task-relevant information (Radulovacki and Adey, 1965; Adey, 1967; Wood et al., 1999; Moita et al., 2003; MacDonald et al., 2011; Aronov et al., 2017). In addition, several studies have provided conclusive data suggesting that hippocampal lesions interfere with learning and memory (Barbizet, 1963; Adey, 1967; Zola and Squire, 2001; Burgess et al., 2002).

In an interesting study using a T-maze discriminative response paradigm, Radulovacki and Adey (1965) showed that during discrimination, averages triggered by a brief tone exhibited periodic θ waves (phase reset), whereas the amplitude and regularity of the waves were reduced or altogether absent when the animal was orienting. The results of Radulovacki and Adey (1965) suggest that the θ phase reset may relate to processes that underlie acquisition and storage of behaviorally relevant information and speculate that “they might underlie the most fascinating continuum in consciousness leading from the immediate past through the present to the immediate future.”

During early training in a classical conditioning paradigm that enabled separation of conditioned responses and purpose-directed responses, the responses evoked by the conditioned stimulus were of low amplitude and usually followed by phase reset of the hippocampal θ (Buzsaki et al., 1979). However, with more training, θ reset decreased and short-latency high-voltage evoked potentials were evoked by the conditioned stimulus. In this condition, orienting activity decreased to the preconditioning level, suggesting changes in oscillation characteristics related to orienting, attentional factors rather than to movements. Although the results suggest that non-spatial information is encoded by a temporal code organized by θ oscillations, how non-spatial information is integrated into memory at the θ timescale is a critical issue that remains to be determined. The experimental analysis of the temporal organization of memory and θ has been limited by experiments that lack the temporal resolution to segregate encoding and retrieval. In human subjects asked to recall previously learned word-object associations, the neural signatures of memory retrieval fluctuate and are time locked to the phase of an ongoing theta oscillation (Kerren et al., 2018). It has been recently reported that in human patients performing a spatial memory task phase locking at the peak of θ preceded eye fixations to retrieved locations, whereas phase locking at the trough of θ followed fixations to novel object-locations, indicating that the hippocampus coordinates memory-guided eye movements (Kragel et al., 2020). These human results strongly suggest that memory encoding retrival is gated by θ-linked neuronal activity.

### Spatial Navigation and Theta

**Figure 4:**
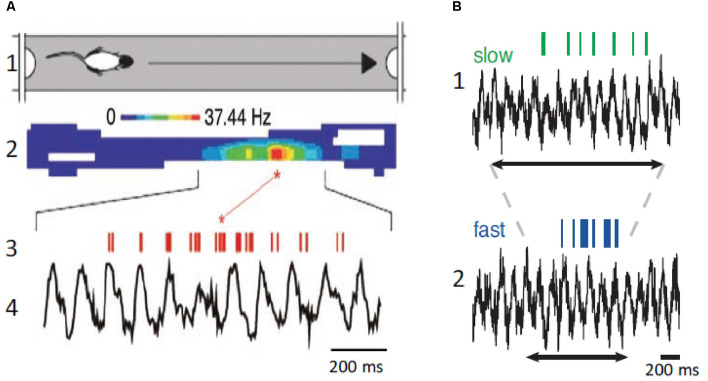
Place neurons and θ. **(A1)** Schematic diagram of rat traveling in the place field. **(A2)** Color coded display of place neuron firing rate in the place field. **(A3,A4)** Place neuron firings (red, upper) and field θ (lower). Note that the neuron displays phase precession and fires at a higher rate at a specific position in the place field. **(B1)** Place cell firing (green, upper) and corresponding field θ (lower) during slow locomotion (mean speed 31 cm/s). **(B2)** Same as **(B1)**, but fast locomotion (mean speed 55 cm/s). The arrows indicate the time it takes the rat to cross the place field. Note that there is no change of field θ and that the neuron displays phase precession at both speeds but fires at a higher rate during the fast trial. **(A)** Modified from Buzsaki and Draguhn (2004); **(B)** modified from Geisler et al. (2007).

The original detailed analysis of the relationship between spatial navigation and hippocampal θ was provided by O’Keefe and Recce (1993). O’Keefe and Recce (1993) discovered that hippocampal neurons began firing at a particular phase of the θ cycle as the rat entered the field and fired at progressively earlier phases of the θ cycle as the rat passed through the neuron’s place field (Figure 4). This phenomenon was called phase precession because the firing phase of the “place cell” was highly correlated with the rat’s spatial location, whereas temporal aspects of behavior were not. Several studies have shown that place cells can represent a position ahead of the animal in the field, suggesting that phase precession can predict the sequence of upcoming positions (Lisman and Redish, 2009; Buzsaki and Moser, 2013). Therefore, the activity in the hippocampus can be encoded as the sequence of action potentials within each θ cycle to signal information about future, present, and past locations and events (Dusek and Eichenbaum, 1998; Lisman and Redish, 2009; Pfeiffer and Foster, 2013; Sugar and Moser, 2019). Accordingly, hippocampal neurons discharge in function of the animal’s location and direction of movement in a given environment suggesting that place cells play a role in navigational planning (O’Keefe and Dostrovsky, 1971; O’Keefe and Recce, 1993; Wilson and McNaughton, 1993; Brown et al., 1998; Zhang et al., 1998; Jensen and Lisman, 2000).

The above-described results imply that knowledge of the speed and direction of movement and landmark information are required to continuously compute the position of the animal during spatial navigation. Information on speed of locomotion is critical to maintain the correct phase relationships between place cell activity and past, present, and future positions. When the animal navigates the place field of a neuron, the number of θ cycles decreases with running speed, but the number of action potentials per θ cycle increases, and the θ phase shifts proportionally, leaving the relationship between action potential phase and spatial position relatively invariant (Geisler et al., 2007; Pfeiffer and Foster, 2013). Place coding in the hippocampus requires sensory inputs providing environmental, self-motion, and place memory information, and effective spatial navigation involves remembering landmarks and goal location to plan navigation paths. Therefore, to continuously compute past, present, and future positions, the hippocampus compares distances and durations through a speed-dependent modulation, and these computations are independent on the behavioral task.

It is noteworthy that John O’Keefe, Edvard Moser, and May Brit Moser were awarded the Nobel Prize in 2014. O’Keeffe for his work on “place cells, ” their relationship with hippocampal θ and spatial navigation, and Edvard and May Britt Moser for the identification of “grid cells” in the entorhinal cortex that are involved in positioning and pathfinding.

## Interaction of Theta with Gamma Rhythms

In the hippocampus, θ and gamma (γ) oscillations are the most prominent rhythms recorded in awake state or during REM sleep (Buzsaki, 2002; Cantero et al., 2003, 2004; Colgin, 2016). These oscillations can be observed in field potential recordings and are thought to transiently link distributed cell assemblies that are processing related information, a function that is important for network processes such as perception, attention, or memory (Singer, 1998; Buzsaki and Draguhn, 2004).

### γ Frequency Bands in the Hippocampus

**Figure 5:**
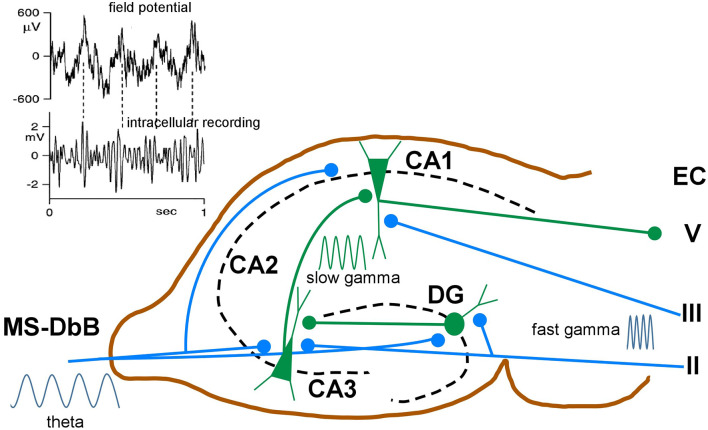
Schematic diagram of neuronal circuits involved in theta-gamma (θ−γ) coupling. Blue traces indicate synaptic inputs from layers II to III of the enthorynal cortex (EC) and from the MS-DbB. The dentate gyrus (DG), CA1, CA3, and EC layer V neuronal connections are also shown. θ−γ coupling in the hippocampus results from the convergence of θ inputs from the MS-DbB and fast γ from the EC. Particularly, θ−γ coupling results in CA1 by the convergence of θ inputs from the MS-DbB and slow γ from the CA3. The inset shows θ−γ interactions in the hippocampus as revealed by the CA1 field activity (upper) and band-pass filtered intracellular γ activity (lower). Note phase locking between both rhythms (θ−γ coupling**)** and the amplitude modulation of the intracellular γ; modified from Penttonen et al. (1998).

Whereas in neocortical network γ oscillations occur across a broad frequency band ranging from 30 to 140 Hz, in the hippocampus, two different frequency bands are usually observed. Slow γ ranges roughly from 25 to 60 Hz and fast γ from 60 to 100 Hz (Bosman et al., 2014). These different frequency bands may route different input streams of information to the hippocampus. Slow γ may facilitate transmission of inputs to CA1 from CA3 (Brun et al., 2002; Schomburg et al., 2014). Fast γ may promote inputs from the entorhinal cortex that transmit ongoing spatial information (Brun et al., 2002; Hafting et al., 2005). Accordingly, fast γ oscillations in CA1 were synchronized with fast γ in medial entorhinal cortex, and slow γ oscillations in CA1 were coherent with slow γ in CA3 (Colgin et al., 2009). The firing properties of hippocampal neurons exhibited in each case may reflect different functions: memory retrieval during slow γ and memory encoding during fast γ oscillations (Figure 5).

### θ–γ Coupling During Memory Task Performance

As indicated above, hippocampal θ activity is involved in forming new episodic memories, especially in encoding of location and time or the context of events (Lisman et al., 2017). During these active states, γ oscillations are prominent and also participate in memory formation (Buzsaki and Draguhn, 2004). γ oscillations are prominent in the entorhinal–hippocampal network during a variety of memory tasks in different species (Montgomery and Buzsáki, 2007; Sederberg et al., 2007; Jutras et al., 2009; López-Madrona et al., 2020). These authors suggest that successful memory performance requires coupling of γ rhythms to particular phases of the hippocampal θ (termed θ–γ coupling). Hippocampal θ–γ coupling was observed during spatial memory processing (Penttonen et al., 1998; Buzsaki and Moser, 2013) and has been shown to support the induction of LTP (Jensen and Lisman, 1996; Buzsaki, 2002; Vertes, 2005).

θ–γ coupling is modulated during exploration and memory-guided behaviors. In mice solving a mnemonic task, CA1 γ oscillations were more strongly phase locked to θ than in control periods (Tort et al., 2008). Similarly, rats learning to associate contexts with the location of food reward show an increase in θ–γ coupling during the learning progression (Lisman and Jensen, 2013). In a word recognition paradigm in humans, θ–γ coupling was selectively enhanced when patients successfully remembered previously presented words (Mormann et al., 2005). In agreement with that, Axmacher and colleagues, using intracranial EEG recordings showed θ–γ coupling in the hippocampus during working memory retention in a working memory task, and the strength of this coupling predicted individual working memory performance (Axmacher et al., 2010).

In rodents, 20–40 Hz oscillations in CA1 became more tightly locked to the θ phase as animals learned odor–place associations, suggesting that θ–γ coupling may play an important role in cued memory retrieval (Igarashi et al., 2014). In another study, γ power in CA1 increased in a delayed spatial alternation task when animals needed to remember which side to choose (Takahashi et al., 2014). θ–γ coupling was mainly observed during episodes of both active wake and REM sleep, with the highest level of coupling observed during REM sleep (Bandarabadi et al., 2019). Taken together, these results support the hypothesis that θ–γ coupling facilitates transfer of spatial information from the enthorinal cortex to CA1 (Brun et al., 2002; Hafting et al., 2005; Colgin et al., 2009).

It has been reported that inputs from the enthorinal cortex and CA3 arrive in CA1 at specific phases of the θ cycle (Hasselmo et al., 2002; Colgin et al., 2009). LTP in CA1 is most easily induced at a particular θ phase, which corresponds to the phase when enthorinal cortex input is maximal (Huerta and Lisman, 1995). Activating γ-modulated cell assemblies at a particular θ phase may allow the network to produce a more powerful output by ensuring that distributed cells fire closely in time (Bragin et al., 1997; Tort et al., 2008). θ–γ coupling may allow the hippocampal–entorhinal network to temporally organize sequences of events within each θ cycle, raising the possibility that θ–γ interactions are a critical component of mnemonic operations (Bragin et al., 1997). θ–γ coupling leads to a precise temporal coordination of spikes of multiple neurons and is therefore likely to contribute to circuital functions such as phase coding or spike-timing-dependent plasticity (Markram et al., 1997; Abbott and Nelson, 2000).

### Theta–Gamma Coupling in Degenerative Pathologies

Taken together, the above results strongly suggest that θ–γ coupling is vital in learning and memory processes, and consequently, its loss may be at the base of pathologies. Working memory deficits are common among individuals with Alzheimer’s dementia (AD) or mild cognitive impairment (MCI). The origin of these deficits has long been thought to be due to hippocampal and enthorinal cortical dysfunctions, although atrophy of MS-DbB also occurs in AD (Cantero et al., 2020). AD and MCI patients demonstrate the lowest level of θ–γ coupling in a verbal working memory task in comparison with healthy participants (Goodman et al., 2018). Similar findings have been observed in animal models of AD, which show a decreased θ–γ coupling (Zhang et al., 2016; Bazzigaluppi et al., 2018) that arises before amyloid beta accumulation (Goutagny and Krantic, 2013; Iaccarino et al., 2016). An impairment of θ–γ coupling that increases paralleling the progression of the MCI has been recently reported in human patients (Musaeus et al., 2020). These findings suggest that θ–γ coupling is critical for proper cognitive functioning and may therefore serve as a progression marker in degenerative diseases.

Place cells in the hippocampus fire action potentials in specific spatial locations (O’Keefe and Dostrovsky, 1971), whereas grid cells in the medial entorhinal cortex fire in a highly organized spatial pattern across an environment (Fyhn et al., 2004; Hafting et al., 2005). Patients with AD and other dementia-spectrum disorders exhibit profound disruption in spatial navigation and memory, even at very early stages of the disease (Hort et al., 2007; Allison et al., 2016). At a pathological level, misfolded tau deposition typically occurs first in the entorhinal cortex and hippocampus (Rubio et al., 2008; Cantero et al., 2011; Llorens-Martin et al., 2014) and reduce grid cell activity (Pooler et al., 2014; Ridler et al., 2020). These data correlate with human imaging studies, which suggest deficits in grid-cell-like activity in the entorhinal cortices of people at genetic risk of developing AD (Kunz et al., 2015).

## Concluding Remarks

In the brain, information is represented by the activity of ensembles of neurons rather than by single cells, coordinating their activity to support complex cognitive processes and creating functional neural networks (Singer, 1998). An efficient way to assure transient synchronization between neuronal ensembles is the entrainment of neuronal groups into oscillatory activities. Oscillations may facilitate neuronal synchronization and synaptic plasticity, playing a key role in long-range communication between brain regions. In this review, we underscore that the θ rhythm results from complex interactions between circuital and intrinsic properties of MS-DbB and hippocampal neurons and that it plays a crucial role in cognitive processes such as learning and memory, and in the control of complex behaviors.

## Author Contributions

AN and WB conceived and wrote all aspects of this review. All authors contributed to the article and approved the submitted version.

## Conflict of Interest

The authors declare that the research was conducted in the absence of any commercial or financial relationships that could be construed as a potential conflict of interest.
